# Adverse childhood experiences and health risk behaviours among adolescents and young adults: evidence from India

**DOI:** 10.1186/s12889-023-15416-1

**Published:** 2023-03-21

**Authors:** Chanda Maurya, Priya Maurya

**Affiliations:** 1 Department of Survey Research and Data Analytics, International Institute for Population Sciences, Mumbai, Maharashtra 400088 India; 2 Department of Population and Development, International Institute for Population Sciences, Mumbai, Maharashtra 400088 India

**Keywords:** Early sexual debut, Negative gender attitude, Stressful childhood events, Substances use, Suicidal thoughts, Violent behaviour

## Abstract

**Background:**

Adverse childhood experiences (ACEs) are traumatic and stressful events that occur in childhood. These experiences at home, school, or in the community may damage the cognitive health and emotional skills of children and adolescents.

**Objective:**

The present study examines the association between Adverse childhood experiences and risky health behaviour indicators while controlling other background characteristics among boys and girls. This study also assesses outcomes in the aggregate to estimate the impact of cumulative adversity on various risky health behavioural factors among boys and girls among adolescents and young adults (age group 13–23) in India.

**Data and methods:**

Data were drawn from the second wave of the “Understanding the lives of adolescents and young adults (2018–2019)” survey. Bivariate and logistic regression analysis were conducted to fulfill the objective.

**Results:**

The findings show that nearly 30% of boys and 10% of girls had violent behaviour. Substance use prevalence was much higher among boys (34.11%) than girls (6.65%). More boys had negative gender attitudes. The majority of the study participants had multiple ACEs. Boys who experienced more than three or more childhood adversity had two times higher odds (OR: 2.04; CI: 1.01–4.16) of the early sexual debut, while the same figure for girls was thirteen times (OR: 13.13; CI: 3.95–43.69) than their male counterparts.

**Conclusion:**

The study findings underlined the need for implementing outcome-oriented approaches to adolescents’ health care and behavioural risks. Therefore, identifying and intervening with adolescents and young adults who are at the highest risk of engaging in risky behaviors early in life may reduce the risk of these behaviors persisting into adulthood. In order to avoid health risk behavior in later stages among adolescents and young adults, policymakers need to focus on ACEs as risk factors and take action to reduce this burden. A potential model could be to create awareness among family members, caregivers, and communities to be more empathetic toward the children.

**Supplementary Information:**

The online version contains supplementary material available at 10.1186/s12889-023-15416-1.

## Introduction

The childhood years, from prenatal to late adolescence and early adulthood, are “building block” years for the basis of intelligence and skill development, self-motivation, social behavior, health and adult relationships, which extend into adulthood [1, 2]. Some level of stress and adversity is a normal part of healthy human development. However, exposure to frequent stressful events without protective factors can result in negative health outcomes [1]. Adverse childhood experiences (ACEs) are traumatic and stressful events that occur in childhood before a child reaches the age of 18. It includes all types of direct and indirect abuse, neglect such as experiencing or witnessing violence, growing up with substance-abusive family members, incarceration of parents, parental separation, sibling or other family members, and suicidal incidence in the household as well as in the community [1, 3, 4]. Evidence also suggests that adverse experiences at home, school, or in the community may damage the cognitive health and emotional skills of children and adolescents [1, 5]. These childhood experiences also undermine their stability, sense of safety, and bonding among children [6]. According to the report of Centers for Disease Control and Prevention (CDC), nearly 62% of adults from the United States of America experienced atleast one type of ACE before the age of 18, and about one in six reported that they had experienced more than three types of ACEs [1]. A study from India done by Fernandes et al. (2021) reported that one in two young people has child mistreatment ACEs and family-level ACEs [2].

ACEs can burden economic costs in the form of healthcare spending, loss of employee productivity, social services, and judicial expenditure [5, 7]. According to a recent estimate, global cost for the burden of violence against children is 2% of the global GDP at the lowest level and it goes up to 8% of the global GDP at the highest level in the year 2013 [5]. ACEs and health risk behaviours (HRBs) are also associated with increased comorbid conditions, early mortality, premature death and increased prevalence of the leading causes of death in adulthood [8, 9]. Many studies have found links between ACEs and long-term health outcomes, including cancer and cardiovascular diseases [8–10]. A shred of literature has also identified that adolescents who are the victim of adverse events are at greater risk of health risk behavior such as engaging in substance abuse, drug use, suicide attempts, sexually transmitted infections, risky sexual behavior, poor mental and physical health outcome, which leads to health disparity over the lifespan [11–15].

Research demonstrates that those who grew up experiencing inter-parental violence are more likely to have externalizing and internalizing behavior problems, trauma symptoms, own perpetration and victimization of violence, dating violence, hopelessness, psychological adjustment problems, and low self-esteem [16–20]. Further, the belief in gender norms and conceptions affects boys and girls differently. For instance, girls are more prone to domestic violence, which leads to internalizing disorders such as depression and anxiety, whereas it affects boys from violent behavior to perpetrating violence [21, 22].

Earlier studies focused on single ACEs mentioned that single predictors of ACEs did not account for a large amount of variance in health outcomes [9, 10]. Moreover, youth exposed to multiple types of maltreatment had a significantly higher chance of depressive disorders [23], substances use problems [24], and poor physical health [25] in comparison to those exposed to a single type. Cumulative Risk Theory also postulates that greater levels of adversity were associated with outcomes in a dose-dependent manner, such as multiple adverse exposures will result in poorer outcomes than single-event exposures [26, 27].

Research has shown that ACEs increase the risk of poor health-related outcomes in later life and most studies discussed the effect of ACEs on mental health, depression, and physical health. Also, the early onset of HRBs envisages their persistence into the later year of life [43]. Evidence demonstrates that ACEs are more common in low and middle-income countries due to lack of limited resources and fewer social and healthcare services [10]. Moreover, owing to the paucity of data, less is known about how ACEs are associated with HRBs in adolescence and early adulthood in the Indian context when many risky health behaviour problems often emerge. Identifying and treating the risk factors that are central to the development of health risk behaviours is pivotal to intervening with vulnerable populations such as adolescents and young adults who have adverse childhood experiences. Therefore, to fill the gaps in the literature in the Indian context, the current research has two objectives. First, to examine the association between adverse childhood experiences and risky health behaviour indicators while controlling with other background characteristics among boys and girls. Second, we assess outcomes in the aggregate to estimate the impact of cumulative adversity on various risky health behavioural factors among boys and girls in the age-group 13–23 years in India. All the analysis is segregated by gender as boys and girls have different kinds of exposure to different risk factors.

## Data and method

### Data

The present study utilized data from the second wave of the “Understanding the lives of adolescents and young adults (UDAYA)” survey conducted by the Population Council under the supervision of the Ministry of Health and Family Welfare, Government of India [28]. The survey is longitudinal in nature and was conducted in two Indian states, namely, Uttar Pradesh and Bihar. The wave-1 of survey was conducted in 2015-16 and a follow-up survey was conducted three years later in 2018-19. The survey collected detailed information on family, community environment, media, assets acquired in adolescence, and quality of transitions to young adulthood indicators.

The UDAYA survey adopted a multi-stage systematic sampling design to provide the estimates for states and urban and rural areas. For each sub-group of the adolescents, the required samples were determined at 920 younger boys, 2,350 older boys, 630 younger girls, 3,750 unmarried older girls, and 2,700 married older girls in each state. Information related to biomarkers was gathered from all younger adolescents and a sub-sample of older adolescents. To achieve the required samples, approximately 36,000 households were covered in each state [28]. A total of 150 (PSUs) visited each state to conduct interviews in the required number of households. As rural and urban areas are treated as independent sampling domains, therefore, drew sample areas independently for each of these domains. The 150 PSUs were divided equally into rural and urban areas. Within each sampling domain, a multi-stage systematic sampling design was adopted [28]. The 2011 census list of villages and wards served as the sampling frame for selection of the villages and wards in rural and urban areas, respectively. This list was stratified using four variables: region, village/ward size, the proportion of the population belonging to scheduled castes and tribes as well as female literacy. For household selection in rural areas, three stages and in urban areas four stages sampling design was adopted. In rural areas, villages were selected by using probability proportional to size (PPS) sampling. In urban areas, firstly 75 wards were selected systematically with probability proportional to size, and then from each wards, after arranging CEBs according to their administrative number, one CEB was selected randomly. To ensure the size of the CEBs, CEBs with less than 500 households merged with the nearest one. A complete mapping and household listing operation were carried out in each selected PSUs. Based on the list of the household list, first the PSUs were divided into two nearly equal segments and one segment was randomly chosen for performing interviews of females and the other for interviews of males. The number of household interviews to be conducted was fixed at 90 in the male segment and 150 in the female segment in each PSU in order to achieve our targeted sample of unmarried boys and girls. Households to be interviewed were selected with equal probability from the list using systematic sampling. The details of sampling are provided in the report [28]. The effective sample size for Uttar Pradesh and Bihar in the first wave was 10,350 and 10,350 adolescents aged 10–19 years, respectively [29]. Moreover, in wave-2 (2018–2019), the study interviewed the participants who were successfully interviewed in 2015–2016 and consented to be re-interviewed. After excluding the respondents who gave an inconsistent response to age and education in the follow-up survey (3%), the final follow-up sample covered 4428 boys and 11,864 girls, with a rate of follow-up 74% for boys and 81% for girls [29]. The substantial sample size for this study was adolescents and young adults aged 13–23 years (boys- 4,221 and girls- 5,987) and was unmarried at both time of the survey.

### Variable description

#### Outcome variables:

The present study has five outcome variables namely violent behaviour, substances use, negative gender attitude, early sexual debut, and suicidal thoughts.

#### Key explanatory variables:

The present study has five key explanatory variables, namely substances use by family members, inter-parental violence, physical abuse, sexual abuse and gender discrimination. Details of the study variable were presented in Supplementary Table 1.

#### Other explanatory variables:

On the basis of previous evidence which has an impact on ACEs and HRB, individual and household level factors were considered as other covariates in the present study. Age group was recoded as 13–19 years and 20–23 years. Current schooling was recoded as no and yes. Co-reside with both parents was recoded as no and yes. Mother’s education was coded as illiterate and literate. Caste was recoded as Schedule caste/Schedule Tribes (SC/ST) and non-SC/ST (including other backward castes and general castes). Religion was recoded as Hindu and Non- Hindu. Wealth Index was divided as poor, middle and rich. Place of residence was recoded as urban and rural. State was recoded as Uttar Pradesh and Bihar.

### Statistical analysis

Descriptive statistics (weighted percentage and unweighted sample) were used to assess the characteristics of the adolescents and young adults included in the study. Bivariate analysis looked at the unadjusted association between outcome variables (violent behaviour, substances use, negative gender attitude, early sexual debut and suicidal thoughts) and explanatory variables. Multivariate logistic regression models were run to calculate adjusted odds ratios that indicated whether certain subgroups of adolescents and younger adults were more or less likely to have adverse childhood experiences and whether or not the experiences predicted the likelihood that adolescents and younger adults would have violent behaviour, substances use, negative gender attitude, early sexual debut, suicidal thoughts. Further, logistic regression analysis was used for the association between multiple ACEs and violent behavior, substance use, negative gender attitude, early sexual debut, and suicidal thoughts. All models were adjusted for all other individual and household-level characteristics and segregated by gender of the respondents. Results were presented as an adjusted odds ratio (AOR) with 95% confidence interval (CI). All the statistical analysis was performed using STATA 14 and MS Excel.

## Result

### Characteristics of the study population

Characteristics of the study population are presented in Table 1. Almost 71% of boys were adolescents, while the same prevalence for girls was 64%. Nearly 37.5% of boys and 49.2% of girls were currently not in school. Around one-third of boys (30.8%) and girls (33.7%) had a literate mothers. Nearly 16.9% of the respondents were living with both their parents. Nearly a third-fourth of respondents belonged to non-SC/ST social groups. The majority were from the Hindu religion. About 30.39% of boys and 26.58% of girls were from poor wealth quantile households. The majority of the respondents were rural residents.

**Table 1:** Socio-demographic characteristics of respondents, 2018-19

Characteristics	Boys		Girls		
	Unweighted sample	Weighted percentage	Unweighted sample	Weighted percentage	
**Age Group (in years)**					
13–19	2,987	71.51	3,858	64.1	
20–23	1,234	28.49	2,129	35.9	
**Current schooling**					
No	1,533	37.51	2,647	49.24	
Yes	2,688	62.49	3340	50.76	
**Mother’s education**					
Illiterate	2,711	69.23	3,725	66.3	
literate	1,510	30.77	2,262	33.7	
**Co-residence with both parents**					
No	740	16.97	1,086	17.15	
Yes	3,481	83.03	4,901	82.85	
**Caste**					
SC/ST	1,012	26.44	1,203	22.81	
Non-SC/ST	3,209	73.56	4,784	77.19	
**Religion**					
Hindu	3,537	84.42	4,390	75.79	
Non-Hindu	684	15.58	1,597	24.21	
**Wealth Index**					
Poor	1,032	30.39	1,240	26.58	
Middle	862	22.39	1,106	20.48	
Rich	2,327	47.22	3,641	52.95	
**Place of residence**					
Urban	1,933	17.39	2,901	19.44	
Rural	2,288	82.61	3,086	80.56	
**State**					
Uttar Pradesh	2,185	67.83	3,476	75.61	
Bihar	2,036	32.17	2,511	24.39	
**Total**	**4,221**		**5,987**		

### Adverse childhood experiences and health risk behaviour among adolescents and young adults

The percentage of different types of childhood adversity experienced and health risk behaviours among adolescents and young adults are presented in Table 2. About a third-fourth of respondents reported that at least one member in their family was substances users. One-fourth of the girls and one-fifth of the boys experienced interparental violence. Physical abuse prevalence was higher among boys (58.94%) than girls (35.91%). About 7% of boys and 13% of girls experienced gender discrimination. About 6.2% of girls were victims of sexual violence, whereas the same prevalence for boys was 1.67%. Further, nearly 30.22% of the boys and 9.62% of the girls had violent behaviour. Substance use prevalence was much higher among boys (34.11%) than girls (6.65%). More boys (84.79) had negative gender attitudes compared to girls (68.02). About 4.55% of the boys were sexually active before age eighteen, while the same prevalence for girls was 1.37%. Suicidal thoughts prevalence was higher among girls (5.05%) than boys (2.19%).

**Table 2:** Percentage distribution of adolescents and young adults by adverse childhood experiences (2015-16) and health risk behaviour, 2018-19

Variables	Boys		Girls	
	Sample	Percent	Sample	Percent
**Adverse childhood experiences**				
Substance use by family member	2,997	73.14	4,148	73.57
Inter-parental violence	746	19.25	1,389	24.7
Physical abuse	2,443	58.94	2,014	35.91
Gender discrimination	293	7.29	696	12.45
Sexual abuse	64	1.67	442	6.2
**Health risk behaviours**				
Violent behavior	1,388	30.22	621	9.62
Substances use	1,477	34.11	439	6.65
Negative gender attitude	3,462	84.79	3,738	68.02
Early sexual debut	155	4.55	79	1.37
Suicidal Thoughts	117	2.19	353	5.05
**Total (N)**	**4,221**		**5,987**	

### Prevalence of health risk behaviours by background characteristics among adolescents and young adults

Table 3 represents the prevalence of health risk behaviours among adolescents and young adults by background characteristics. Boys and girls whose family members were substances users reported a higher prevalence of violent behaviour (boys: 30.3%; girls: 10.2%), substances use (boys: 37%; girls: 7.1%), negative gender attitudes (boys: 85.9%, girls: 71.2%), early sexual debut (boys: 5.2%; girls: 1.6%) as well as having thoughts about suicide (boys: 2.1%; girls: 5.3%). Risky health behaviour was more prevalent among those who witnessed interparental violence. Victims of physical abuse had a higher prevalence of violent behaviour (boys: 33.7%; girls: 11.5%), substances use (boys: 33.9%; girls: 6.5%), negative gender attitudes (boys: 88.9%; girls: 73.9%), sexually active before eighteen years (boys: 4.4%; girls: 2.2%) and suicidal thoughts (boys: 2.2%; girls: 5.6%).

**Table 3:** Prevalence of selected health risk behaviours by type of adverse childhood experiences and other background characteristics among adolescents and young adults, 2018-19

Variables	Violent behaviour		Substances Use		Negative gender attitude		Early sexual debut		Suicidal Thoughts	
	Boys	Girls	Boys	Girls	Boys	Girls	Boys	Girls	Boys	Girls
	N (%)	N (%)	N (%)	N (%)	N (%)	N (%)	N (%)	N (%)	N (%)	N(%)
**Substances used by family members**										
No	383(30.01)	161(8.05)	350(26.34)	128(5.37)	949(81.83)	981(59.24)	32(2.85)	12(0.62)	32(2.46)	95(4.44)
Yes	1,005(30.29)	460(10.18)	1,127(36.97)	311(7.11)	2,513(85.88)	2,757(71.18)	123(5.17)	67(1.64)	85(2.09)	258(5.27)
**Inter-parental violence**										
No	1,125(29.29)	452(9.21)	1,165(32.65)	343(6.62)	2,824(84.37)	2,770(65.77)	118(4.08)	36(0.75)	94(2.16)	244(4.56)
Yes	263(34.11)	169(10.86)	312(40.27)	96(6.77)	638(86.57)	968(74.89)	37(6.5)	43(3.27)	23(2.31)	109(6.53)
**Physical abuse**										
No	505(25.16)	358(8.55)	625(34.49)	289(6.74)	1,427(83.22)	2,343(64.71)	66(0.75)	35(0.75)	51(2.18)	222(4.73)
Yes	883(33.74)	263(11.52)	852(33.85)	150(6.5)	2,035(85.89)	1,395(73.94)	89(3.27)	44(3.27)	66(2.2)	131(5.61)
**Sexual Abuse**										
No	25(30.91)	50(9.36)	28(40.59)	41(6.95)	57(88.45)	267(64.93)	8(15.17)	32(9.5)	5(7.34)	39(6.55)
Yes	1,363(30.21)	571(9.63)	1,449(34)	398(6.63)	3,405(84.73)	3,471(68.23)	147(4.37)	47(0.83)	112(2.1)	314(4.95)
**Gender discrimination**										
No	1,296(29.97)	548(9.81)	1,361(33.66)	402(6.81)	3,221(84.75)	3,233(66.85)	136(4.3)	66(1.23)	110(2.27)	306(4.78)
Yes	92(33.41)	73(8.29)	116(39.91)	37(5.58)	241(85.41)	505(76.29)	19(7.73)	13(2.36)	7(1.17)	47(6.95)
**Age group (in years)**										
13–19	1,116(34.43)	453(10.71)	850(27.95)	288(6.38)	2,496(85.97)	2,550(72.25)	55(1.9)	37(0.93)	76(1.77)	230(5.22)
20–23	272(19.65)	168(7.66)	627(49.58)	151(7.15)	966(81.83)	1,188(60.47)	100(11.18)	42(2.15)	41(3.25)	123(4.74)
**Current schooling**										
No	424(25.12)	256(9.62)	868(56.51)	190(6.86)	1,362(90.44)	1,962(78.31)	109(8.99)	54(1.93)	63(3.00)	199(7.01)
Yes	964(33.28)	365(9.61)	609(20.67)	249(6.45)	2,100(81.41)	1,776(58.05)	46(1.88)	25(0.83)	54(1.71)	154(3.15)
**Co-reside with both parents**										
No	258(33.26)	120(10.63)	299(38.47)	98(7.03)	615(87.15)	698(70.01)	30(4.38)	23(2.42)	38(3.71)	70(5.55)
Yes	1130(29.6)	501(9.41)	1178(33.22)	341(6.58)	2847(84.31)	3,040(67.61)	125(4.58)	56(1.15)	79(1.88)	283(4.94)
**Mother’s education**										
Illiterate	895(29.33)	420(10.71)	1,022(35.82)	269(6.71)	2368(88.37)	2703(75.92)	111(4.94)	66(1.84)	72(2.06)	229(5.01)
literate	493(32.22)	201(7.47)	455(30.27)	170(6.54)	1094(76.74)	1,035(52.48)	44(3.65)	13(0.45)	45(2.48)	124(5.13)
**Caste**										
SC/ST	310(27.03)	152(11.49)	430(58.11)	79(41.89)	852(5.39)	803(86.78)	58(6.52)	34(3.05)	40(2.61)	88(6.34)
NON-SC/ST	1078(31.36)	469(9.06)	1,047(68.68)	360(31.32)	2610(7.03)	2,935(84.08)	97(3.84)	45(0.87)	77(2.04)	265(4.67)
**Religion**										
Hindu	1,176(30.73)	474(9.45)	1,223(33.54)	295(6.01)	2,881(84.97)	2,588(64.67)	128(4.61)	66(1.66)	103(2.36)	270(5.16)
Non-Hindu	212(27.46)	147(10.14)	254(37.25)	144(8.66)	581(83.84)	1,150(78.53)	27(4.19)	13(0.47)	14(1.28)	83(4.69)
**Place of residence**										
Urban	675(32.77)	298(9.68)	708(34.74)	244(8)	1484(76.2)	1,490(50.6)	51(3.14)	24(0.8)	67(3.14)	193(5.77)
Rural	713(29.68)	323(9.6)	769(33.98)	195(6.33)	1978(86.6)	2,248(72.23)	104(4.84)	55(1.51)	50(1.99)	160(4.87)
**Wealth Index**										
Poor	340(31.59)	173(11.84)	399(36.53)	76(6.2)	938(91.64)	1,020(84.07)	43(4.94)	30(2.19)	22(1.6)	74(5.37)
Middle	281(29.99)	116(9.67)	332(38.12)	74(6.17)	777(89.33)	836(78.77)	35(4.84)	19(1.62)	25(2.22)	80(6.17)
Rich	767(29.44)	332(8.48)	746(30.66)	289(7.07)	1747(78.24)	1882(55.81)	77(4.15)	30(0.86)	70(2.56)	199(4.45)
**State**										
Uttar Pradesh	639(28.35)	295(8.54)	750(33.14)	248(6.81)	1,791(83.59)	2,201(66.9)	103(5.02)	46(1.37)	61(2.26)	184(4.58)
Bihar	749(34.16)	326(12.95)	727(36.17)	191(6.18)	1,671(87.33)	1,537(71.49)	52(3.55)	33(1.38)	56(2.05)	169(6.49)
**Total**	**1,388(30.22)**	**621(9.62)**	**1,477(34.11)**	**439(6.65)**	**3,462(84.79)**	**1,882(68.02)**	**155(4.55)**	**79(1.37)**	**117(2.19)**	**353(5.05)**

### Determinants of health risk behaviours among adolescents and young adults

Table 4 depicts the multivariate logistic regression analysis estimate for health risk behavior among adolescents and young adults. Substances used by family members were significantly associated with increased odds of violent behaviour [boys- AOR: 1.19, CI: 1.02–1.38; girls- AOR: 1.28, CI: 1.05–1.57], substances use [boys- AOR: 1.38, CI: 1.17–1.62; girls- AOR: 1.21, CI: 0.97–1.52] and negative gender attitudes [boys- AOR: 1.18, CI: 0.98–1.41; girls- AOR: 1.28, CI: 1.12–1.45] than their counterparts. On considering familial ACEs, boys who reported witnessing interparental violence had higher odds for substance use behaviors [AOR: 1.29, CI: 1.08–1.55] and girls had greater odds of early sexual debut [AOR: 2.21, CI: 1.31–3.72] than their counterparts. Girls who experienced interparental violence were 35% more likely to have suicidal thoughts than those who did not experience interparental violence. Boys [AOR: 1.34, CI: 1.17–1.54] and girls [AOR: 1.41, CI: 1.17–1.69] who had been a victim of physical abuse were significantly at greater risk of violent behavior than those who had not been a victim of physical abuse. Further, Girls who experienced physical violence were more likely to have negative gender attitudes [AOR: 1.28, CI: 1.12–1.46] and an early sexual debut [AOR: 1.68, CI: 1.00-2.82]. Victimization of sexual abuse was significantly associated with early sexual debut and suicidal thoughts among both boys and girls. Girls who experienced gender discrimination in their childhood were more likely to have negative gender attitudes [AOR: 1.22, CI: 1.00-1.48] than those who did not experience gender discrimination. The probability of involvement in the early sexual debuts was 72% higher among boys who experienced gender discrimination.

**Table 4:** Logistic regression estimates on association between selected health risk behaviour and adverse childhood experiences among adolescents and young adults, 2018-19

Variables	Violent behavior		Substances Use		Negative gender attitude		Early sexual debut		Suicidal thoughts	
	AOR (95% CI)		AOR (95% CI)		AOR (95% CI)		AOR (95% CI)		AOR (95% CI)	
	Boys	Girls	Boys	Girls	Boys	Girls	Boys	Girls	Boys	Girls
**Substances used by family members**										
No®										
Yes	1.19**(1.02 1.38)	1.28**(1.05 1.57)	1.38***(1.17 1.62)	1.21*(0.97 1.52)	1.18*(0.98 1.41)	1.28***(1.12 1.45)	1.24 (0.82 1.9)	1.91*(0.99 3.71)	1.26 (0.81 1.95)	1.14 (0.88 1.48)
**Inter-parental violence**										
No®										
Yes	1.09 (0.92 1.31)	1.01 (0.82 1.24)	1.29***(1.08 1.55)	0.94 (0.73 1.22)	0.98 (0.77 1.24)	1 (0.86 1.17)	1.22 (0.81 1.84)	2.21***(1.31 3.72)	1.19 (0.73 1.94)	1.35**(1.03 1.75)
**Physical abuse**										
No®										
Yes	1.34***(1.17 1.54)	1.41***(1.17 1.69)	1.02 (0.88 1.18)	1.1 (0.88 1.38)	1.03 (0.87 1.23)	1.28***(1.12 1.46)	1.07 (0.76 1.53)	1.68**(1 2.82)	0.99 (0.67 1.47)	1.05 (0.82 1.34)
**Sexual Abuse**										
No®										
Yes	1.31 (0.78 2.21)	1.04 (0.76 1.43)	1.59 (0.91 2.77)	1.31 (0.93 1.84)	1.74 (0.78 3.89)	1.01 (0.81 1.25)	3.69***(1.62 8.39)	7.14***(4.37 11.68)	3.26**(1.24 8.61)	1.36*(0.95 1.95)
**Gender discrimination**										
No®										
Yes	0.9 (0.7 1.17)	0.89 (0.68 1.16)	1.21 (0.93 1.58)	0.71*(0.5 1.00)	0.85 (0.61 1.17)	1.22**(1.00 1.48)	1.72**(1.01 2.9)	0.82 (0.43 1.55)	0.87 (0.39 1.91)	1.03 (0.74 1.44)
**Age group (in years)**										
13–19®										
20–23	0.53***(0.45 0.62)	0.78**(0.64 0.95)	2.05***(1.76 2.39)	0.91 (0.74 1.13)	0.64***(0.53 0.77)	0.65***(0.58 0.74)	3.71***(2.58 5.33)	2.67***(1.62 4.41)	0.96 (0.63 1.45)	0.91 (0.72 1.16)
**Current schooling**										
No®										
Yes	1.29***(1.11 1.49)	1.14(0.95 1.38)	0.27***(0.24 0.50)	1.01(0.82 1.26)	0.48***(0.39 0.59)	0.53***(0.46 0.60)	0.34***(0.23 0.50)	0.58*(0.34 0.98)	0.45***(0.31 0.68)	0.54***(0.43 0.69)
**Co-reside with both parents**										
No®										
Yes	0.81**(0.68 0.96)	0.88 (0.71 1.10)	0.69***(0.57 0.83)	0.72***(0.56 0.91)	1 (0.8 1.26)	0.99 (0.85 1.15)	0.84 (0.55 1.31)	0.43***(0.25 0.73)	0.37***(0.25 0.57)	0.85 (0.64 1.12)
**Mother’s education**										
Illiterate®										
literate	0.93 (0.8 1.08)	0.86 (0.7 1.05)	0.97 (0.83 1.13)	1.04 (0.83 1.3)	0.58***(0.49 0.69)	0.6***(0.53 0.68)	0.97 (0.66 1.43)	0.57*(0.30 1.10)	1.25 (0.83 1.90)	1.03 (0.80 1.33)
**Caste**										
SC/ST®										
NON-SC/ST	1.15*(0.98 1.36)	0.85 (0.68 1.05)	0.69***(0.59 0.82)	1 (0.76 1.31)	1.12 (0.91 1.39)	1.02 (0.87 1.19)	0.55***(0.38 0.81)	0.51**(0.30 0.85)	0.58**(0.38 0.88)	0.85 (0.64 1.11)
**Religion**										
Hindu®										
Non-Hindu	0.91 (0.75 1.10)	0.93 (0.75 1.16)	0.98 (0.8 1.19)	1.34**(1.06 1.70)	1.25*(0.98 1.60)	1.77***(1.52 2.05)	1.1 (0.68 1.78)	0.8 (0.40 1.57)	0.62 (0.34 1.13)	0.78*(0.58 1.03)
**Place of residence**										
Urban®										
Rural	0.99 (0.81 1.20)	0.74**(0.57 0.95)	1.04 (0.85 1.28)	1.05 (0.75 1.46)	1.07 (0.78 1.46)	0.76***(0.62 0.93)	1.11 (0.69 1.79)	0.72 (0.39 1.33)	1.52 (0.84 2.77)	1.26 (0.91 1.76)
**Wealth Index**										
Poor®										
Middle	1.01 (0.84 1.21)	0.69***(0.55 0.87)	0.86 (0.71 1.04)	1.19 (0.88 1.6)	0.46***(0.35 0.59)	0.43***(0.36 0.51)	1.17 (0.75 1.83)	0.53**(0.29 0.98) 1.65*(0.95 2.88) 0.96(0.70 1.32)		
Rich	0.77***(0.67 0.9)	0.85 (0.71 1.03)	0.78***(0.67 0.91)	0.81*(0.65 1.01)	1.29***(1.07 1.55)	1.85***(1.63 2.09)	1.83***(1.25 2.69)	1.45 (0.84 2.52)	0.63**(0.41 0.95)	0.64***(0.5 0.81)
**State**										
Uttar Pradesh®										
Bihar	1.3***(1.14 1.49)	1.56***(1.31 1.85)	1.19**(1.04 1.37)	1.08 (0.88 1.32)	0.99 (0.84 1.17)	1.06 (0.94 1.2)	0.7**(0.49 1)	1.23 (0.75 2.02)	0.96 (0.65 1.41)	1.31**(1.04 1.64)
**Constant**	0.52***(0.38 0.70)	0.15***(0.11 0.22)	0.38***(0.28 0.52)	0.08***(0.05 0.13)	6.01***(4.04 8.92)	1.6***(1.22 2.10)	0.01***(0.00 0.02)	0.01***(0.00 0.02)	0.04***(0.02 0.10)	0.06***(0.03 0.09)

Note: AOR: Adjusted odds ratio; CI: Confidence Interval; ®: Reference category; SC/ST: Scheduled Caste/Scheduled Tribe; *if p *<* 0.05, **if p < 0.01, *** if p < 0.001

### Prevalence and effect of cumulative adverse childhood experiences on risky health behaviours

**Fig. 1:**
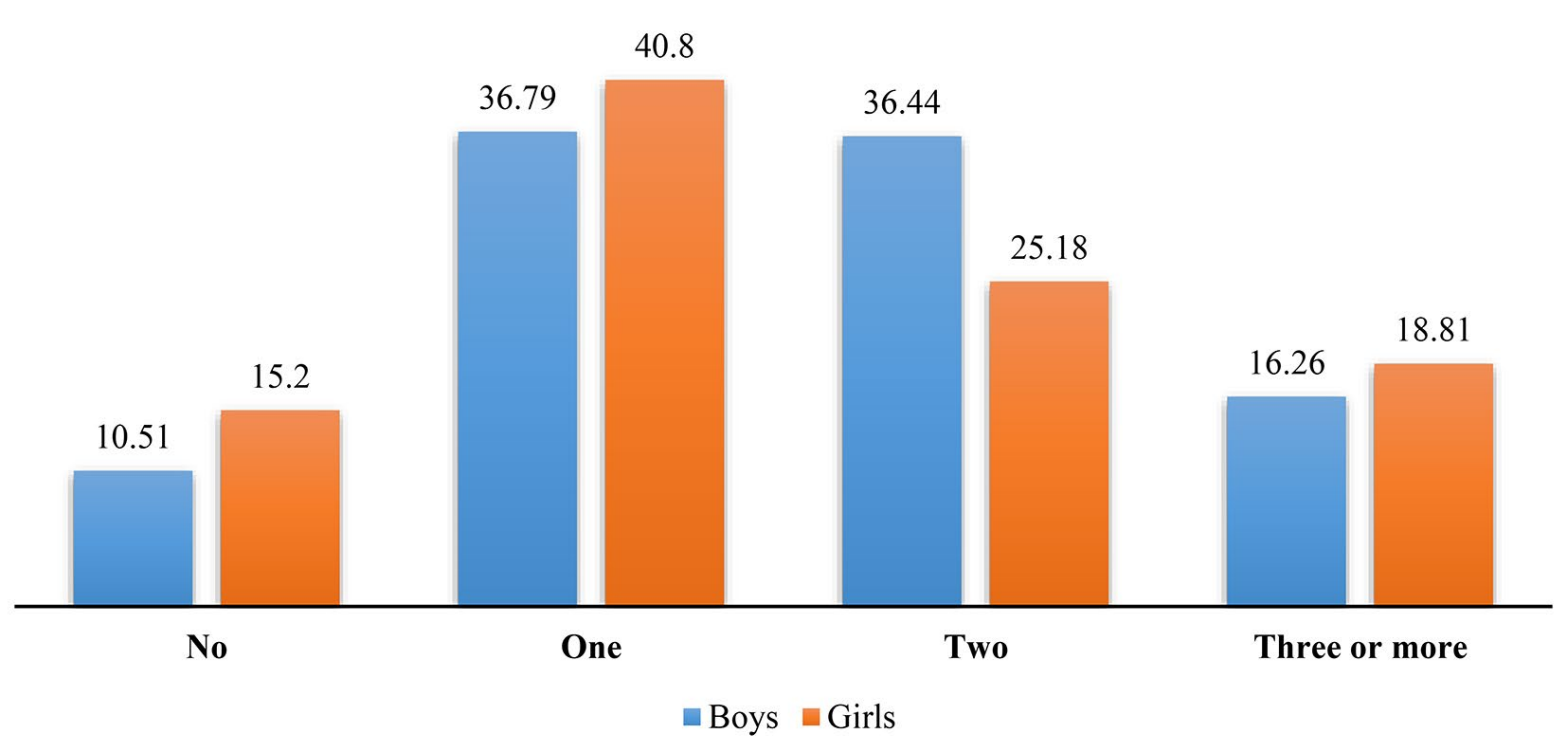
Exposure to multiple adverse childhood experiences among adolescents and young adults, 2018-19

**Fig. 2:**
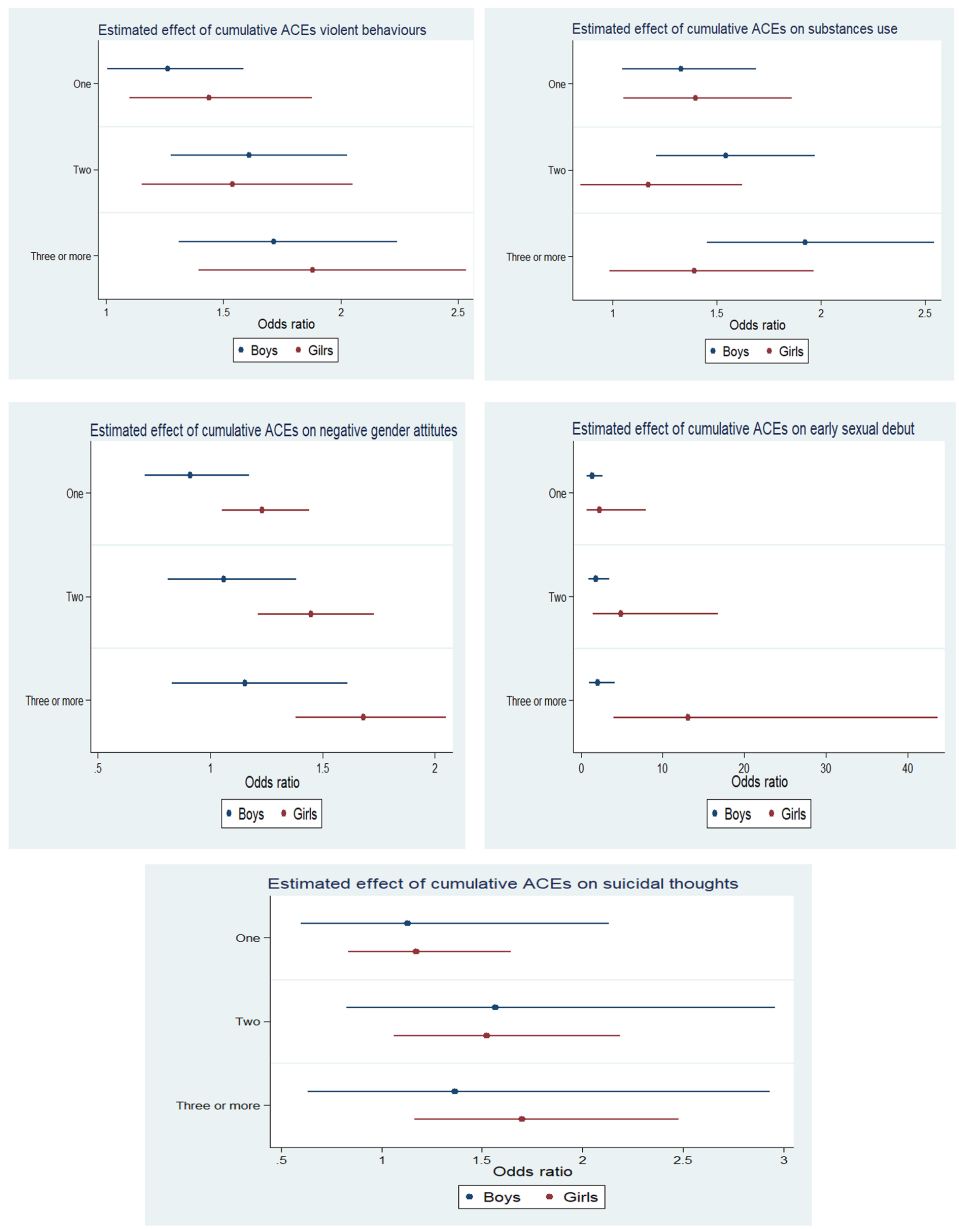
Logistic regression estimates on association between selected health risk behaviour and multiple adverse childhood experiences among adolescents and young adults, 2018-19 Note: OR: Odds ratio; CI: Confidence Interval; ®: Reference category; *if p *<* 0.05, **if p < 0.01, *** if p < 0.001 All the other variables were controlled.

The majority of the study participants had multiple ACEs. Around one in five girls (18.81%) had three or more ACEs, whereas the same prevalence for boys was 16.26% (Fig. 1). Adolescents and younger adults who experienced three or more ACEs had significantly higher odds of risky health behaviors than those with no childhood adversity experience. Gender differences were observed in the magnitude of odds for health risk behaviour. Boys who experienced more than three or more childhood adversity were twice [AOR: 2.04; CI: 1.01–4.16] odds of the early sexual debut, while the same figure for girls was thirteen times [AOR: 13.13; CI: 3.95–43.69] than their counterparts (Fig. 2).

## Discussion

Health risk behaviors, including violent behavior, substance use, early sexual debut and suicidal thoughts, are the leading cause of morbidity and mortality among adolescents and young adults. Adolescents who experience adverse childhood are at higher risk of adopting negative health behavior. ACEs are stressful and traumatic, leading to immediate health hazards and affecting health across the lifespan [1, 9]. Social learning theory also suggests that social behaviour is learned through observation, imitation and modeling [30]. Therefore, understanding the developmental consequences of ACEs on health is important for developing a strength-based model. The current study expands the evidence by demonstrating how ACEs are associated with HRBs among adolescents. Consistent with our hypothesis, single and multiple ACEs have partially related to adverse health risk behavior. However, the strength of association was not consistent across all health risk domains among girls and boys.

The present study findings indicate that substance use by family members and physical violence was the most common type of adverse childhood experience. This is not unusual since India is the second-largest tobacco consumer after China [31]. Physical abuse of children by family members is considered as a normal part of life and quite acceptable in the Indian traditional family system. The conceivable reason for such kind of activity is that it helps improve performance in academics and good behaviour and becomes well-mannered [32, 33]. A study on college students in South India mentioned that around 43% of respondents considered themselves believed that some sort of punishment is necessary to develop good behaviour among children [32]. Sexual abuse generates deep concern for public health worldwide and has also been considered the most severe form of abuse among children [13]. In the present study, 1.67% of boys and 6.2% of girls experienced sexual abuse in childhood. Previous studies from India also reported similar prevalence of different forms of sexual abuse ranging from 2.6 to 14.3% [32]. A systematic review and meta-analysis of 55 studies from 24 countries conducted in 2013 found that the prevalence of child sexual abuse ranges from 8 to 31% among girls and 3–17% among boys [34]. Its traumatic impact leads to substance use, mental illness, suicide, abusive behaviour, teenage pregnancies, and sexually transmitted diseases that deteriorate the physical health of victims [9, 10, 13, 35]. In our study, sexual abuse was higher among girls (6.2%) than boys (1.67%), moreover, physical abuse prevalence was higher among boys than girls. A meta-analytic review also stated that boys are at higher risk of experiencing severe physical abuse, psychological abuse and neglect, whereas girls are more likely to be victims of sexual abuse [15]. Earlier evidence have also mentioned that male victims are less likely to report sexual abuse [2, 36], so the observed gender differences might be related to reporting bias. Therefore, it requires special attention.

Further, this study findings indicate that at least one adverse childhood event was reported by more than one-third of adolescents and was more prevalent among females (40.8%) than males (36.79%). Moreover, overall, ACEs were higher among boys than girls. Similar findings were observed in a cohort study among the minority in the United States [24]. Exposure to different ACEs showed a range of 73.57–1.67%. This prevalence is lower when compared with other studies from India [2, 37]. Kacker et al. (2007) reported that 68.9% of children were exposed to physical abuse; 53% experienced sexual abuse; 48.4% suffered emotional abuse and 70.6% experienced neglect [37]. Similarly, data from “Consortium on Vulnerability to Externalising Disorders and Addictions (cVEDA)” found that more than half of the participants reported child maltreatment and family-level ACEs such as domestic violence [2]. Nevertheless, these differences in prevalence must be explained by the measures taken consider in ACE, sample size and age group of the study population.

The present study yields supportive evidence for the significant association between childhood adversity and poor risky lifestyle habits in later life. In general, the more adverse experience one has faced in childhood, the higher the probability for those individuals to engage in risky lifestyle behaviour, consequently suffer from negative health habits such as violent behaviour, smoking behaviour, early sexual debut and having mental disorders in later age [3, 38, 39]. Our findings suggested that substance use by family members was a significant risk factor for HRB, except for suicidal thoughts among adolescents and young adults. The social learning model also postulated that tobacco, alcohol or drug consumption are learned behaviour from the individuals and surroundings [30, 40]. Sexual abuse was positively associated with suicidal thoughts. This association may be elucidated by the fact that childhood trauma can negatively impact one’s ability to maintain cognitive health, resulting in risky health behaviours [39, 41]. Results indicated that gender discrimination experiences were positively associated with negative gender attitudes among girls. Previous evidence on discrimination asserted that adolescents who have gone through cultural-based stress or discrimination may experience negative gender attitudes and depressive symptoms. Also, these discrimination experiences could lead to long-term psychological maladjustment, worsening their health [38]. A recent study on youth with childhood adversity experiences stated that those with engaged in prosocial peer groups were less likely to indulge in risky behaviour. On the other hand, those who socialized with antisocial peer groups were at higher risk of risky health behaviours [42]. The current study also finds an unclear association between ACEs and selected health risk behaviour such as sexual abuse with violent behaviour, substances use and negative gender attitude. Therefore, these findings may imply that other individual and household level factors have impact on health outcome other than ACEs.

Further, co-occurring maltreatment is very common than single maltreatment [43, 44]. Individuals with history of multiple types of maltreatment were at greater risk of violent behaviour, substance use, early sexual debut and suicidal thoughts and it appears to be a relatively strong dose-response relationship [39, 44]. The cumulative theory posits that if individual experiences more adverse events, health outcomes will be poorer than single event exposure [44–46]. Frequent or co-occurring childhood adversity may increase the harmful consequences of these adverse events to a greater extent [40, 47]. Adverse childhood experiences hamper cognitive development which leads to psychobiological vulnerability and developmental delays. Harmful health behaviour such as substances use maladaptive as a way of coping strategies with external and internal psychological and other challenges that are difficult for the person to manage. Individuals with higher ACEs had greater substance dependency [2, 47].

This study has several limitations. First, the UDAYA data were used for the study which was conducted in two states of the country, which limits the representativeness of our results. Therefore, the findings can’t be generalized at the country level. Second, the ACEs and HRBs were self-reported. Therefore, it is challenging to validate the extent of self-report and might be subject to recall bias. Third, though the study used a number of outcome variables and explanatory variables based on previous literature, however, all potential confounders were not available in the dataset, and for that reason, we were not able to consider them in the study. Fourth, in the present study, only a few ACEs have been studied. Other ACEs such as cyberbullying, harassment, aggressive behaviour, and fighting with peer groups in school which was available in the dataset, were not considered in the present study. Therefore, further research is required for the standardizing evaluation of ACEs and HRBs at the population level.

## Conclusion

Adverse childhood experiences are common and have a massive impact on health and social outcomes. Thus, it has public health challenges with implications for the entire lifespan and every health and well-being domain. Also, multiple risk behaviour and condition often exist together in the same individual, adding cumulative risk for poor health outcomes in later stages. The study findings underlined the need for implementing outcome-oriented approaches to adolescents’ health care and behavioural risks. Therefore, identifying and intervening with adolescents and young adults who are at greater risk of engaging in risky behaviors early in life may reduce the risk of these behaviors persisting into adulthood. In order to avoid health risk behavior in later stages among adolescents and young adults, policymakers need to focus on ACEs as risk factors and take action to reduce this burden. A potential model could be to create awareness among family members, caregivers and communities to be more empathetic toward the children. Also, the decision-maker needs to work towards ensuring the protection of their rights and preventing their exploitation by formulating guidelines and strict laws.

## Electronic supplementary material

Below is the link to the electronic supplementary material.

Supplementary Material 1

## Data Availability

The study utilizes a secondary source of data that is freely available on request through: https://dataverse.harvard.edu/dataset.xhtml?persistentId=doi:10.7910/DVN/ZJPKW5.

## List of abbreviations

**ACEs**: Adverse childhood experiences
**CI**: Confidence interval
**HRBs**: Health risk behaviours
**AOR**: Adjusted Odds Ratio

## References

[1] Centers for Disease Control and Prevention. *Adverse Childhood Experiences - Prevention Strategies*. Atlanta, 2021.

[2] Fernandes GS, Spiers A, Vaidya N, et al. Adverse childhood experiences and substance misuse in young people in India: results from the multisite cVEDA cohort. BMC Public Health. 2021;21:1–13. 34686158 10.1186/s12889-021-11892-5 PMC8539836

[3] Felitti VJ, Anda RF, Nordenberg D, et al. Relationship of childhood abuse and Household Dysfunction to many of the leading causes of death in adults the adverse childhood experiences (ACE) study. Am J Prev Med. 1998;14:245–58. 9635069 10.1016/s0749-3797(98)00017-8

[4] Wiehn J, Hornberg C, Fischer F. How adverse childhood experiences relate to single and multiple health risk behaviours in german public university students: a cross-sectional analysis. BMC Public Health. 2018;18:1–13. 10.1186/s12889-018-5926-3 PMC6090638 30103728

[5] Pereznieto P, Montes A, Langston L et al. *The costs and economic impact of violence against children*. 2014.

[6] Bick J, Nelson CA. Early adverse experiences and the developing brain. Neuropsychopharmacology. 2016;41:177–96. 26334107 10.1038/npp.2015.252 PMC4677140

[7] Tang B, Jamieson E, Boyle M, et al. The influence of child abuse on the pattern of expenditures in women’s adult health service utilization in Ontario, Canada. Soc Sci Med. 2006;63:1711–9. 16793185 10.1016/j.socscimed.2006.04.015

[8] Ozieh MN, Garacci E, Campbell JA, et al. Adverse childhood experiences and decreased renal function: impact on all-cause mortality in U.S. adults. Am J Prev Med. 2020;59:e49–e57. 32690202 10.1016/j.amepre.2020.04.005 PMC7378887

[9] Campbell JA, Walker RJ, Egede LE. Associations between adverse childhood experiences, high-risk behaviors, and morbidity in Adulthood. Am J Prev Med. 2016;50:344–52. 26474668 10.1016/j.amepre.2015.07.022 PMC4762720

[10] Chang X, Jiang X, Mkandarwire T, et al. Associations between adverse childhood experiences and health outcomes in adults aged 18–59 years. PLoS ONE. 2019;14:1–11. 10.1371/journal.pone.0211850 PMC6366931 30730980

[11] Hemmingsson E, Johansson K, Reynisdottir S. Effects of childhood abuse on adult obesity: a systematic review and meta-analysis. Obes Rev. 2014;15:882–93. 25123205 10.1111/obr.12216

[12] Norman RE, Byambaa M, De R et al. The Long-Term Health Consequences of Child Physical Abuse, Emotional Abuse, and Neglect: A Systematic Review and Meta-Analysis.PLoS Med; 9. Epub ahead of print 2012. DOI: 10.1371/journal.pmed.1001349. 10.1371/journal.pmed.1001349 PMC3507962 23209385

[13] Odhayani A, Al, Watson WJ, Watson L. Behavioural consequences of child abuse. Can Fam Physician. 2013;59:831–6. 23946022 PMC3743691

[14] Springer KW, Sheridan J, Kuo D, et al. The long-term health outcomes of childhood abuse: an overview and a call to action. J Gen Intern Med. 2003;18:864–70. 14521650 10.1046/j.1525-1497.2003.20918.x PMC1494926

[15] Wegman HL, Stetler C. A meta-analytic review of the effects of childhood abuse on medical outcomes in adulthood. Psychosom Med. 2009;71:805–12. 19779142 10.1097/PSY.0b013e3181bb2b46

[16] Temple JR, Shorey RC, Tortolero SR, et al. Importance of gender and attitudes about violence in the relationship between exposure to Interparental Violence and the perpetration of Teen dating violence. Child Abus Negl. 2013;37:343–52. 10.1016/j.chiabu.2013.02.001 PMC3670104 23490056

[17] Shen ACT. Long-term effects of interparental violence and child physical maltreatment experiences on PTSD and behavior problems: a national survey of taiwanese college students. Child Abus Negl. 2009;33:148–60. 10.1016/j.chiabu.2008.07.006 19327836

[18] O’Keefe M. The differential effects of family violence on adolescent adjustment. Child Adolesc Soc Work J. 1996;13:51–68.

[19] Haj-Yahia MM. The incidence of witnessing interparental violence and some of its psychological consequences among arab adolescents. Child Abus Negl. 2001;25:885–907. 10.1016/s0145-2134(01)00245-9 11523867

[20] Arriaga XB, Foshee VA. Adolescent dating violence do adolescents follow in their friends’, or their parents’, Footsteps? J Interpers Violence. 2004;19:162–84. 15006000 10.1177/0886260503260247

[21] Sinha A, Chowdhury B, Heuveline P. Physical intimate partner violence in India: how much does childhood socialisation matter? Asian Popul Stud 2022;1–20. 10.1080/17441730.2022.2035921 PMC10656056 37982075

[22] Hosang GM, Bhui K. Gender discrimination, victimisation and women’s mental health. Br J Psychiatry. 2018;213:682–4. 30475196 10.1192/bjp.2018.244

[23] Chapman DP, Whitfield CL, Felitti VJ, et al. Adverse childhood experiences and the risk of depressive disorders in adulthood. J Affect Disord. 2004;82:217–25. 15488250 10.1016/j.jad.2003.12.013

[24] Mersky JP, Topitzes J, Reynolds AJ. Impacts of adverse childhood experiences on health, mental health, and substance use in early adulthood: a cohort study of an urban, minority sample in the U.S. Child Abus Negl. 2013;37:917–25. 10.1016/j.chiabu.2013.07.011 PMC4090696 23978575

[25] Crandall AA, Miller JR, Cheung A, et al. ACEs and counter-ACEs: how positive and negative childhood experiences influence adult health. Child Abus Negl. 2019;96:104089. 10.1016/j.chiabu.2019.104089 31362100

[26] Monnat SM, Chandler RF. Long-term Physical Health Consequences of adverse childhood experiences. Sociol Q. 2015;56:723–52. 26500379 10.1111/tsq.12107 PMC4617302

[27] Richardson S, Carr E, Netuveli G et al. Adverse events over the life course and later-life wellbeing and depressive symptoms in older people.Int Psychogeriatrics. Epub ahead of print 2020. DOI: 10.1017/S1041610220003373. 10.1017/S1041610220003373 33050971

[28] Santhya KG, Acharya R, Pandey N et al. *Understanding the lives of adolescents and Young adults (UDAYA) in Uttar Pradesh, India*, www.popcouncil.org (2016).

[29] Santhya K. *UDAYA, Adolescent Survey, Bihar and Uttar Pradesh, 2018–19*. Epub ahead of print 2020. DOI: 10.7910/DVN/ZJPKW5.

[30] Trujillo EM, Suárez DE, Lema M, et al. How adolescents learn about risk perception and behavior in regards to alcohol use in light of social learning theory: a qualitative study in. Int J Adolesc Med Heal. 2015;27:3–9. 10.1515/ijamh-2014-0003 24864297

[31] GATS factsheet. Global adult Tobacco Survey-2. Maharashtra: Mumbai; 2016.

[32] Bhilwar M, Upadhyay RP, Rajavel S, et al. Childhood experiences of physical, emotional and sexual abuse among College students in South India. J ofTropical Pediatr. 2015;61:329–38. 10.1093/tropej/fmv037 26130618

[33] Runyan ADK, Prevention I, Hill C, et al. International Variations in Harsh Child Discipline. Pediatrics. 2015;126:e701–11. 10.1542/peds.2008-2374 20679301

[34] Barth J, Bermetz L, Heim E, et al. The current prevalence of child sexual abuse worldwide: a systematic review and meta-analysis. Int J Public Health. 2013;58:469–83. 23178922 10.1007/s00038-012-0426-1

[35] Musa S, Peek-Asa C, Jovanović N, et al. Association of adverse childhood experiences and health risk behaviors among young adults visiting a regional primary healthcare center, Federation of Bosnia and Herzegovina. PLoS ONE. 2018;13:1–14. 10.1371/journal.pone.0194439 PMC5875750 29596442

[36] Mlouki I, Bouanene I, Sioud I, et al. Impulsivity mediates the impact of early life adversity on high risk behaviors among tunisian adolescents. Prev Med Reports. 2021;23:101424. 10.1016/j.pmedr.2021.101424 PMC8190465 34150480

[37] Kacker L, Varadan S, Kumar P. Study on child abuse: INDIA 2007. New Delhi; 2007.

[38] Davis AN, Carlo G, Schwartz SJ, et al. The Longitudinal Associations between discrimination, depressive symptoms, and Prosocial Behaviors in U.S. Latino/a recent immigrant adolescents. J Youth Adolesc. 2016;45:457–70. 26597783 10.1007/s10964-015-0394-x PMC11194831

[39] Novais M, Henriques T, Vidal-alves MJ. When problems only get bigger: the impact of adverse childhood experience on Adult Health. Front Psychol. 2021;12:1–12. 10.3389/fpsyg.2021.693420 PMC8318698 34335410

[40] Smith MA. Social Learning and Addiction. Behav Brain Res. 2021;398:112954. 33053384 10.1016/j.bbr.2020.112954 PMC7719575

[41] Daines CL, Hansen D, Novilla MLB, et al. Effects of positive and negative childhood experiences on adult family health. BMC Public Health. 2021;21:1–8. 33820532 10.1186/s12889-021-10732-w PMC8022401

[42] Yoon D. Peer-relationship patterns and their association with types of child abuse and adolescent risk behaviors among youth at-risk of maltreatment. J Adolesc. 2020;80:125–35. 32088414 10.1016/j.adolescence.2020.02.008

[43] Ramiro LS, Madrid BJ, Brown DW. Adverse childhood experiences (ACE) and health-risk behaviors among adults in a developing country setting. Child Abus Negl. 2010;34:842–55. 10.1016/j.chiabu.2010.02.012 20888640

[44] Garrido EF, Weiler LM, Taussig HN. Adverse childhood Experiences and Health-Risk Behaviors in Vulnerable Early Adolescents. J Early Adolesc. 2018;38:661–80. 29861530 10.1177/0272431616687671 PMC5976451

[45] Jia Z, Wen X, Chen F, et al. Cumulative exposure to adverse childhood experience: depressive symptoms, suicide intensions and suicide plans among senior high school students in Nanchang city of China. Int J Environ Res Public Health. 2020;17:1–13. 10.3390/ijerph17134718 PMC7369761 32630073

[46] Sunitha S, Gururaj G. Health behaviours & problems among young people in India: cause for concern & call for action. Indian J Med Res. 2014;140:185–208. 25297351 PMC4216492

[47] Douglasa KR, Chana G, Gelernterb J, et al. Adverse childhood events as risk factors for Substance Dependence: partial mediation by Mood and anxiety Disorders. Addict Behav. 2010;35:7–13. 19720467 10.1016/j.addbeh.2009.07.004 PMC2763992

