# Correction: Adverse childhood experiences and health risk behaviours among adolescents and young adults: evidence from India

**DOI:** 10.1186/s12889-024-20828-8

**Published:** 2024-12-09

**Authors:** Chanda Maurya, Priya Maurya

**Affiliations:** 1 Department of Survey Research and Data Analytics, International Institute for Population Sciences, Mumbai, 400088 Maharashtra India; 2 Department of Population and Development, International Institute for Population Sciences, Mumbai, 400088 Maharashtra India

**BMC Public Health (2023) 23:536**

10.1186/s12889-023-15416-1

The original publication of this article contained an incorrect footnote in table 4 and figure 2 due to a typo. The incorrect and correct information is listed in this correction article. The original article has been updated.

Incorrect

*** if p < 0.1

Correct

*** if p < 0.001

