# Supplementary material for: Adverse childhood experiences and health risk behaviours among adolescents and young adults: evidence from India
Source: BMC Public Health. 2023 Mar 21;23:536. doi: 10.1186/s12889-023-15416-1 (PMC10031876; doi:10.1186/s12889-023-15416-1)
Supplement: Supplementary file 1 — Supplementary Material 1 [file 12889_2023_15416_MOESM1_ESM.docx]

**Supplementary table 1: Descriptions of the study variables.**

| **Variables** | **Description** |
| --- | --- |
| ***Outcome variables*** | |
| Violent behaviour | Violent behaviour was assessed through a direct question to the respondents "have you been involved in a physical fight with others, including beating, pulling hair and slapping?" Respondents who reported no violent behaviour were categorized as "no", while those who answered in affirmation were categorized as "yes". |
| Substances uses | Substances uses were assessed through the question related to alcohol, tobacco, and smoking. Respondents were asked "have you ever consumed tobacco products (eg., smoke cigarette, eat paan, gutka), alcohol and drugs (ganja, charas, brown sugar, cocaine or locally made substance)?" Those who consumed any one of the above-mentioned items were coded as "yes" and other "no." |
| Negative gender attitude | Negative gender attitude was measured using nine questions regarding the gender attitude among girls and boys. (i) Is it more important to educate boys than girls? (ii) Should boys do as much domestic work as girls? (iii) Is it wrong for a girl to have male friends? (iv)Do girls like to be teased by boys? (v) Should girls be allowed to decide when they want to marry? (vi) Should husband alone/mainly decide how household money is to be spent? (vii) Is it better for girls to get married early than completing at least class 12? (viii) Are giving the kids a bath and feeding the kids women's responsibility only? (ix) There are times when a wife deserves to be beaten by her husband. All the above responses were recoded on a binary scale. The scale of range 0-9 was generated by using the additive method in Stata 14. Then the response variable was recoded as no "for not having any negative gender attitude" and yes "for having negative gender attitude." |
| Early sexual debut | The early sexual debut was determined through the question, "how old were you when you first had sexual intercourse?" Those who reported before age eighteen were considered as early sexual debut and recoded as "yes" and for others "no." |
| Suicidal thoughts | Suicidal thoughts was recoded as "yes" "if the respondent seriously considers attempting suicide" and for others "no." |
| ***Key explanatory variables*** | |
| Substances use by family member | Substances use by family member was determined through the questions "Does anyone in your family consume tobacco products/ alcohol or drugs?" The response was recoded as "no" and "yes." |
| Inter-parental violence | Inter-parental violence was assessed through a direct question from the respondents "has your father ever beaten your mother". The response was coded as "no" and "yes." |
| Physical Abuse | Physical abuse was determined through the question "Have you been physically hurt (for example, beaten) by your father or mother from the time you turned 10 years old?" The response was coded as "no" and "yes." |
| Sexual abuse | Sexual abuse was assessed through the question from girls, "Has any boy/men ever touched you in a bad way when you didn't want to be touched" and from boys, "Has anyone ever tried to force you to have sex with themor had sexual intercourse with you by using physical force and/or threat?" The response was coded as "no" and "yes |
| Gender discrimination | Gender discrimination was measured by three questions regarding the gender discrimination among boys and girls at wave 1. (i) for boys: there were more or better quality of food and for girls there were less or bad quality of food than their counter parts. (ii) more packet money for boys and less for girls (iii) made to study more/ in a better quality school for boys and Made to study less/ in a poor quality school for girls. All the questions marks on binary scale: no and yes. Then the scale and 0-3 were generated by the additive methods and categorized as 0 for "no" and else for "yes." |
